# Assessing the Effects of Cytoprotectants on Selective Neuronal Loss, Sensorimotor Deficit and Microglial Activation after Temporary Middle Cerebral Occlusion

**DOI:** 10.3390/brainsci9100287

**Published:** 2019-10-22

**Authors:** Julius V. Emmrich, Sohail Ejaz, David J. Williamson, Young T. Hong, Sergey Sitnikov, Tim D. Fryer, Franklin I. Aigbirhio, Heike Wulff, Jean-Claude Baron

**Affiliations:** 1 Stroke Research Group, Department of Clinical Neurosciences, University of Cambridge, Cambridge CB2 1QQ, UK; julius.emmrich@charite.de (J.V.E.); drsohailejaz@gmail.com (S.E.); s.l.sitnikov@gmail.com (S.S.); 2 Department of Neurology and Center for Stroke Research, Charité—Universitätsmedizin Berlin, Corporate Member of Freie Universität Berlin, Humboldt-Universität zu Berlin, and Berlin Institute of Health, 10117 Berlin, Germany; 3 Wolfson Brain Imaging Centre, Department of Clinical Neurosciences, University of Cambridge, Cambridge CB2 1QQ, UK; djw84@cam.ac.uk (D.J.W); yth20@cam.ac.uk (Y.T.H.); tdf21@wbic.cam.ac.uk (T.D.F.); fia20@medschl.cam.ac.uk (F.I.A.); 4 Department of Pharmacology, School of Medicine, University of California Davis, Davis, CA 95817, USA; hwulff@ucdavis.edu; 5 Department of Neurology, Sainte-Anne Hospital, INSERM U1266, Paris University,75014 Paris, France

**Keywords:** selective neuronal loss, microglial activation, PET, ischemic stroke, KCa3.1, TRAM-34, reperfusion injury

## Abstract

Although early reperfusion after stroke salvages the still-viable ischemic tissue, peri-infarct selective neuronal loss (SNL) can cause sensorimotor deficits (SMD). We designed a longitudinal protocol to assess the effects of cytoprotectants on SMD, microglial activation (MA) and SNL, and specifically tested whether the KCa3.1-blocker TRAM-34 would prevent SNL. Spontaneously hypertensive rats underwent 15 min middle-cerebral artery occlusion and were randomized into control or treatment group, which received TRAM-34 intraperitoneally for 4 weeks starting 12 h after reperfusion. SMD was assessed longitudinally using the sticky-label test. MA was quantified at day 14 using in vivo [^11^C]-PK111195 positron emission tomography (PET), and again across the same regions-of-interest template by immunofluorescence together with SNL at day 28. SMD recovered significantly faster in the treated group (*p* = 0.004). On PET, MA was present in 5/6 rats in each group, with no significant between-group difference. On immunofluorescence, both SNL and MA were present in 5/6 control rats and 4/6 TRAM-34 rats, with a non-significantly lower degree of MA but a significantly (*p* = 0.009) lower degree of SNL in the treated group. These findings document the utility of our longitudinal protocol and suggest that TRAM-34 reduces SNL and hastens behavioural recovery without marked MA blocking at the assessed time-points.

## 1. Introduction

In acute stroke, due to proximal artery occlusion, mechanical thrombectomy (MT) can rescue the tissue at risk of infarction, i.e., the ischemic penumbra [1]. However, >50% of MT-treated patients do not recover to an independent life [2]. Novel adjunctive therapies are therefore needed to improve outcome after MT [3]. Specifically, brain cytoprotectants [4] may prevent detrimental processes that occur post-reperfusion and affect the rescued penumbra [5,6].

One such process is selective neuronal loss (SNL), documented in rodents subjected to temporary middle cerebral artery occlusion (MCAo) as patchy neuronal loss without extracellular matrix disruption nor death of other brain or vascular cells (see [7] and [8] for review). Importantly, neocortical SNL affects sensorimotor behaviour in rats [9,10], and has also been documented in the rescued penumbra in stroke patients [11,12,13,14]. Microglia activation (MA) triggered by signalling molecules released by injured neurons is closely associated with SNL [15]. MA occurs in the infarct itself but also the salvaged penumbra, and progresses over time, peaking around day 10–15 [7,16]. It is widely assumed that early MA (up to ~day 7) is detrimental via release of neurotoxic cytokines, whereas it subsequently takes on more pro-repair roles [15,17,18]. Early MA therefore stands as a strong candidate for exacerbating peri-infarct neuronal death after reperfusion. This hypothesis is supported by the striking topographical congruence and quantitative correlation found between SNL and MA in rodent models [9,19,20,21,22,23]. However, only intervention studies will make it possible to determine causality. Crucially, if MA exacerbates SNL, blocking it should improve functional outcome. Although the effects of MA blockers on lesion volumes have been extensively studied [15], whether blocking MA is able to curb SNL has not been addressed so far. 

The present study aimed to evaluate the feasibility of longitudinally assessing the effects of pharmacological agents on sensorimotor deficit (SMD), microglial activation and neuronal loss induced by transient focal cerebral ischemia in rodents. Specifically, MA was measured twice in the same subject and in the same brain regions, first in vivo using [^11^C]-PK11195, a validated ligand for post-stroke MA [23], and then two weeks later using post-mortem immunofluorescence (IF). The degree of ultimate SNL was also determined again in the same brain regions using IF. Both MA and SNL were evaluated across the entire hemisphere using a regions-of-interest (ROI) template. Finally, sensorimotor performance was assessed longitudinally from stroke onset until euthanasia. Major advantages of this protocol are (i) enhanced statistical power due to the longitudinal design; (ii) comprehensive assessment of MA and SNL across the whole hemisphere (as opposed to within selected areas only); and (iii), reduced variance (as compared to studying different sets of subjects) thanks to obtaining all the data in the same subjects. 

To test this protocol, we used TRAM-34, a pharmacological agent that potently and selectively blocks the calcium-activated potassium channel KCa3.1 [24]. KCa3.1 is expressed in microglia [25], where it is involved in migration, respiratory burst, inflammatory cytokine and nitric oxide production [25,26,27], as well as in microglia-mediated neuronal killing in organotypic slice cultures [26,28]. Accordingly, TRAM-34 started 12 h post reperfusion improves behavioural deficit following temporary MCAo in rodent models [28,29]. 

## 2. Material and Methods

### 2.1. Overall Study Design

**Figure 1:**
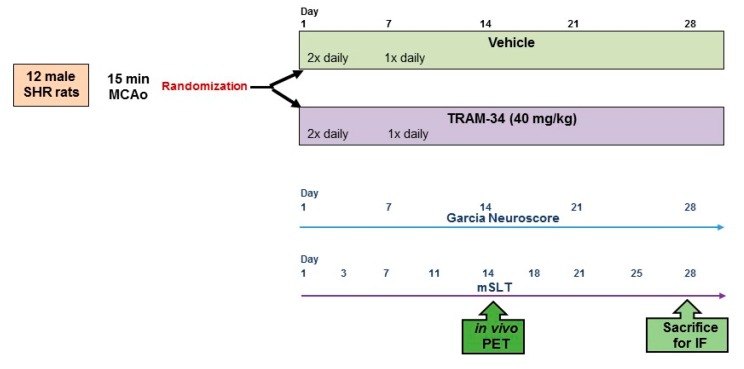
Experimental flow-chart. Abbreviations: SHR: spontaneously hypertensive rats; MCAo: middle-cerebral artery occlusion; mSLT: modified sticky-labelled test; PET: positron emission tomography; IF: immunofluorescence.

As the rat strain, we used spontaneously hypertensive rats (SHRs), which closely mimic the standard stroke population and in which we previously reported the presence of SNL and MA following 15-min distal MCAo [9]. Twelve adult male SHRs underwent distal 15-min tMCAo and were then randomized to receive TRAM-34 or vehicle treatment for 4 weeks. Rats were tested for sensorimotor deficit three times a week for 28 days, underwent [^11^C]-PK11195 PET on day 14, were euthanized on day 28 after the last behavioural assessment, and then their brains were collected for quantitative IF for both MA and SNL. The experimental flow-chart is shown in Figure 1. All data analyses, i.e., behavioural, IF and PET, were carried out blinded to the subject’s group allocation. Analysis of the PET and IF data was carried out in the same set of ROIs according to our previously published methodology [21,23,30], allowing within-subject assessment of changes. 

### 2.2. Animals

This study was approved by the University of Cambridge Ethical Review Panel. In accordance with the legislation of the UK Animals Scientific Procedures Act 1986, the Ethical Review Board required that the study be designed so as to keep the number of animals used to a minimum, yet sufficient to obtain meaningful results. Accordingly, we estimated that this pilot, proof-of-principle study should involve 12 subjects, 6 in the TRAM-34 treated group and 6 in the control group. All procedures were performed on anaesthetized ~3–6-month-old male SHRs (300–400 g body weight). Arterial blood pressure is known to be already significantly elevated by 3 months of age in SHRs [31,32].

Subjects were randomized into the two groups immediately after completion of the MCAo procedure (described in Section 2.4), and all animals used in this study are reported. There was no mortality from the surgery.

### 2.3. Anaesthesia

Experiments were performed in freely breathing animals. Anaesthesia was induced with 4% isoflurane administered in a 0.3 L/min O_2_ and 0.7 L/min N_2_O mix and maintained with 2% isoflurane during surgical procedures. Body temperature was monitored with a rectal probe and maintained at 37.0 ± 0.5 °C using a heated pad throughout all surgical procedures. Blood oxygen saturation and heartbeat were continuously monitored using a pulse-oximeter and remained within normal ranges throughout. 

### 2.4. Middle Cerebral Artery Occlusion (MCAo)

Microclip distal temporary MCAo (md-tMCAo) was performed using the method described by Buchan et al., [33] as implemented in our laboratory and detailed previously [9,19,20,21,23,34,35,36]. The MCA clip was applied onto the left MCA and removed after 15 min and the wound was closed. MCA reperfusion, visually evident on clip removal as immediate reflow distal to the clip [21], was present in all 12 rats of this study. Briefly, the left common carotid artery (CCA) was isolated through a ventral midline incision on the neck and a loose ligature of 4–0 silk suture was placed around it. With the rat positioned onto its right flank, a 2.5 cm skin incision perpendicular to and bisecting a line between the lateral canthus of the right eye and the external auditory canal was then made, and the underlying temporalis muscle excised to reveal the base of the skull. Under direct visualization, the underlying temporalis muscle was excised and craniectomy was performed under saline irrigation to expose the left MCA through a 2 mm burr hole drilled 2 to 3 mm rostral to the fusion of the zygomatic arch with the squamosal bone. The dura was retracted to visualize the MCA at a position where it crosses the inferior cerebral vein, which lies within the rhinal fissure. A micro-aneurysm clip (No 1, Codman, Sundt AVM, USA) was placed on the MCA proximal to the point where it crosses the inferior cerebral vein in the rhinal fissure, and then the left CCA was permanently ligated.

### 2.5. Treatment Groups

The treated group received TRAM-34 40 mg/kg i.p. twice daily for 1 week starting 12 h post-MCAo, then once daily for 3 weeks, as per the previously published protocol [29]. The control group received vehicle (i.e., neutral oil 812, Miglyol, Spectrum Chemicals) only. TRAM-34 was synthesized in our laboratory as previously described [24].

### 2.6. Behavioural Testing

Behavioural testing was carried out by experienced investigators, blinded to the animal’s group. Animals were single-housed on a 12-h light/dark cycle and had free access to water and standard rodent chow. Training/testing was performed in the light phase and animals were left in their housing cages during sessions. Animals received daily handling for at least 4 days before baseline testing to ensure accurate behavioural results. Garcia’s Neuroscore was administered the day before surgery and at postoperative days 1, 7, 14, 21, and 28. The modified sticky label test (mSLT) was performed one day before surgery and postoperatively on days 1, 3, 7, 11, 14, 18, 21, 25, and 28, i.e., three times a week. 

Garcia’s Neuroscore consists of motor, sensory, reflex, and observational tests to evaluate neurological deficits following MCAo in rats [37]. It is scored on a scale from 3 to 18 (normal: 18; maximal deficit: 3), i.e., the lower the score the worse the deficit.

Subtle sensorimotor dysfunction following MCAo was assessed using the mSLT, as previously described [38,39]. This test is sensitive to subtle ischemic damage even when the Neuroscore is normal [10,40] and, contrary to the standard version, uses a non-removable tape. As a result, non-stroked rats spend most of the 30 s sessions trying to remove it, with no habituation effect over time [38,39]. Following stroke, sensorimotor deficits make rats spend less time attending to the tape than normally [40]. Deficits on the SLT are thought to reflect a mix of subtle sensorimotor impairments including hemi-sensory neglect and extinction, forepaw motor deficit and impaired somatosensory integration [38,40,41,42]. A small patch of paper tape (2.5 cm long, 1.0 cm wide) was wrapped around the animal’s wrist contralateral to the ischemic insult such that the tape sticks to itself and that the fingers protrude from the sleeve formed. The rat was placed in its home cage and the time spent attending to the stimulus, be it using the teeth or contralateral paw, was recorded. Animals are given five sessions per day, each observation period lasting for a maximum of 30 s. After each trial the tape was removed and animals given a resting time of ≥3 min. mSLT performance was calculated by dividing the time attending to the stimulus by 30 s, expressing the fraction of the observation period that the animal spends attending to the tape. The best two ratios on each day were averaged. The results of the final day of pre-surgery training served as baseline for assessment of post-MCAo performance. 

### 2.7. [^11^C]-PK11195 PET

At day 14 post-MCAo, all 12 rats underwent [^11^C]-PK11195 applying the same procedures as previously reported [23], save for using an improved scanner. 

#### 2.7.1. PET Scanning Procedure

At day 14, animals were re-anaesthetized as above, had a venous cannula inserted in the tail vein and were positioned in a purpose-built plastic frame incorporating ear bars and a bite bar at the centre of a microPET Focus-220 scanner (Concorde Microsystems, Knoxville, TN, USA). High specific activity (~180 MBq/nmol) [^11^C]-PK11195 was injected i.v. as a 1 mL bolus. The mass of administered PK11195 was purposely limited in order to avoid receptor saturation and the activity was similar for all rats (~2 nmol/kg). Under 1.5% isoflurane, PET data was acquired in list mode (350–650 keV energy window, 6 ns timing window) for 90 min and binned into the following time frames: 6 × 10 s + 3 × 20 s + 6 × 30 s + 10 × 1 min + 10 × 2 min + 11 × 5 min. Prior to [^11^C]-PK11195 injection, a 9 min singles-mode ^68^Ge transmission scan was acquired for attenuation correction.

#### 2.7.2. MRI Acquisition

Magnetic resonance imaging (MRI) was carried out on day 14 of reperfusion immediately after PET scanning, and included T2-weighted and diffusion weighted image (DWI) sequences, as previously detailed [9]. Given the previously reported 100% recanalization rate on MR angiography (MRA) in temporary distal microclip MCAo as implemented in our lab [9,19]. MRA was not performed in this study. Images were acquired using a 4.7T Bruker BioSpec 47/40 system (Bruker, BioSpin GmbH, Ettlingen, Germany) with a 2 cm surface coil used for signal reception. Structural imaging was performed with a T2-weighted RARE sequence (TR/TE 3500/36 ms, ETL 8, slice thickness 1 mm, in-plane resolution 0.156 mm). DWIs were acquired using an EPI sequence (TR/TE 3000/35 ms, 35 directions b = 1000 s/mm^2^, slice thickness 1.5 mm, in-plane resolution 0.312 mm).

#### 2.7.3. PET Data Post-Processing

The basic procedures have been detailed previously [23]. PET images were reconstructed using Fourier rebinning followed by 2D filtered backprojection with a ramp filter cut-off at the Nyquist frequency. Each image array was 128 × 128 × 95, with voxel dimensions 0.95 × 0.95 × 0.80 mm. The PET data were corrected for randoms, dead time, normalization, attenuation, and sensitivity. For each subject, the mean PET image was manually co-registered to the T2-weighted MRI using MPI Tool software (Max Planck Institute, Cologne, Germany).

Parametric images of [^11^C]-PK11195 non-displaceable binding potential (BP_ND_) were produced using the basis function version of the simplified reference tissue model (SRTM) [43]. The ipsilateral cerebellum, manually defined on a symmetric SHR MRI template [23] and inverse warped to each individual T2-weighted MRI using SPM5 (www.fil.ion.ucl.ac.uk/spm), was used as the reference tissue [44,45]. Each individual T2-weighted MRI was warped to the MRI template using SPM5 and this transformation was applied to the co-registered BP_ND_ map to bring it to template space for regional analysis. Please note that this method may generate negative BP_ND_ values, which simply represent lower specific binding than in cerebellum.

### 2.8. Immunofluorescence (IF)

On day 28, rats were perfusion-fixed, and the brain was removed, fixed, coronally sectioned and stained with NeuN and isolectin-B4 (IB4), a reliable marker of activated microglia, especially following ischemic stroke, as described in detail elsewhere [19,46,47,48,49]. Briefly, the brain was removed from the skull and kept in paraformaldehyde for 24 h, transferred to 30% sucrose solution (0.1 M phosphate buffer saline (PBS), pH 7.4) for at least 3–4 days, and cut into 40-μm-thick coronal sections on a sliding microtome (Leica). Sections were collected from across the MCA territory, i.e., from the level of the forceps minor of the corpus callosum to the visual cortex and the superior colliculi according to Paxinos and Watson [50]. Sections were then incubated for 2 h at room temperature in PBS plus 5% normal goat serum and 0.3% Triton X-100 (all Sigma). Sections were stained with anti-NeuN antibody (Millipore Bioscience Research Reagents, 1:500) and biotinylated IB4 (Sigma, 1:500) overnight at 4 °C, washed, and incubated with goat anti-mouse-Cy3 antibody (Jackson ImmunoResearch, 1:150) for 2 h at room temperature. Sections were washed, mounted on gelatine-covered slides, dried for 15 min on a heating block (40 °C) and coverslipped using FluorSave reagent (Calbiochem). 

### 2.9. Histopathological Evaluation of Ischemic Damage

As detailed previously [21], eight coronal sections spanning the MCA territory were selected at the following locations relative to the bregma: 2.70 mm, 1.00 mm, −0.26 mm, −0.92 mm, −2.12 mm, −0.14 mm, −4.52 mm and −6.04 mm. A qualitative visual assessment of the presence of tMCAo-induced lesions was first carried out for each rat by displaying matched NeuN and IB4 sections, blind to the stroke side and the subject’s group. 

The MCAo-induced changes were then quantified as detailed elsewhere [30]. Briefly, a template of 44 cytoarchitectonically defined regions of interest (ROIs) covering the grey matter of the whole MCA territory (per hemisphere: 39 cortical ROIs, four caudate/putamen ROIs and one thalamic ROI), extracted from the Paxinos and Watson atlas for the eight selected coronal sections, was mapped onto the eight digitized sections [21,23]. For each ROI, remaining neurons and activated microglia cells were automatically quantified using Image J, a software that allows automated cell counting methodology (U.S. National Institutes of Health, Bethesda, MD, USA; http://imagej.nih.gov/ij/), as previously detailed [30]. The relative change of the number of NeuN-labelled neurons between corresponding ipsi- and contralesional ROIs was expressed for each ROI as percentage of the difference in number of NeuN-labelled cells present in the affected-side ROI relative to the unaffected-side mirror ROI, divided by the latter. Then, for each rat, a whole hemisphere average cell loss was derived by calculating the weighted mean hemispheric percent difference across all 44 ROIs, i.e., taking into account their surface area.

IB4 has strong and selective affinity for activated, as opposed to resting, macrophages/microglia, but also binds to perivascular and endothelial cells [49,51]. For each ROI, to identify only activated microglia and avoid inadvertently counting other cell types or artefacts, IB4-labelled particles were selectively quantified on binary images based on average (±1 s.d.) value for size and circularity of activated microglia, and the number of IB4-labelled particles on the unaffected side was subtracted from that on the affected side, as detailed in our previous article [30]. Similar to the above for NeuN, for each rat a weighted-mean MA index was then derived by averaging the inter-hemispheric positive cell differences across all 44 ROIs, normalizing each ROI by its area. 

### 2.10. PET Data Analysis

An automated method for data analysis was applied to the entire parametric image data set without knowledge of the subject’s group until after statistical analysis was complete. The eight coronal cuts of the MRI template matching as precisely as possible the eight Paxinos sections used for the IF image analysis were first selected. Then, the same template of 44 symmetrical ROIs used for IF data analysis was applied to the corresponding slices of the co-registered BP_ND_ maps to obtain the mean BP_ND_ value for each ROI. For each pair of symmetrical ROIs, the (affected–unaffected) difference in BP_ND_ was calculated. A mean ± SD (affected–unaffected) BP_ND_ difference across all 44 ROIs, weighted by the ROI volume, was then calculated for each rat and then across the 6 rats of each group. 

### 2.11. Statistical Analysis

The modified Sticky Label Test (mSLT) data were analysed using one-way repeated measures ANOVA (rm-ANOVA) to assess the main effect of Time within each group. If significant, post-hoc multiple comparisons were then conducted by Holm-Bonferroni corrected *t*-tests to assess each time-point relative to baseline. A two-way rm-ANOVA was then used to compare TRAM-34-treated animals to the control group, with *post hoc* multiple comparisons tests if a significant Group effect or Time x Group interaction emerged.

Regarding the IF data, within-subject analyses were performed first, assessing the effects of tMCAo by comparing across the 44 ROIs the affected to the unaffected hemisphere for NeuN and IB4 (paired *t*-tests). Between-group differences in weighted-mean interhemispheric difference in NeuN or IB4 staining were then tested, using non-parametric Mann-Whitney tests, given the small samples (i.e., *n* = 6 vs. *n* = 6). 

A similar approach was used for the PET data. First, within-subject analyses compared the (affected–unaffected) BP_ND_ differences to neutral (i.e., zero) across the 44 ROIs, using t-tests. Then, the weighted-mean (affected–unaffected) BP_ND_ differences were compared between the two groups using Mann-Whitney test.

Results were considered statistically significant if two-tailed *p* was < 0.05.

## 3. Results

Twelve adult male SHRs underwent 15 min distal MCAo and were randomized into a vehicle-treated control group and a TRAM-34-treated group. All 12 SHR rats entered into the study completed the protocol without any complication or early death until the 28-day protocol termination. MA was assessed in vivo using [^11^C]-PK111195 PET imaging at day 14 and by IF on day 28 together with SNL. The modified sticky label test was administered 3 times a week to assess sensorimotor dysfunction. 

### 3.1. Behaviour

As expected, given the short MCAo duration, the Garcia neuroscore was zero, i.e., normal, in all animals of both groups at all time points, indicating no detectable neurological impairment. 

**Figure 2:**
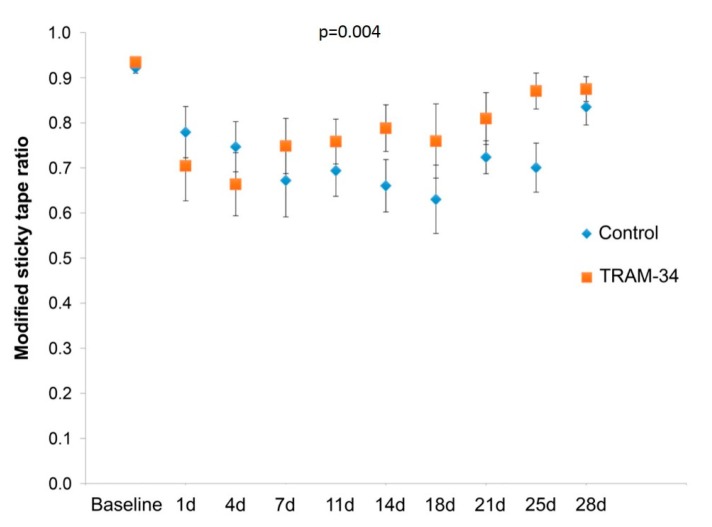
Affected-side daily modified Sticky Label Test (mSLT) data (mean and SEM) for the control and TRAM-34 groups, showing clear-cut initial effects of MCAo on sensorimotor performance in both groups and faster recovery in the treated than the control group. The statistical analysis confirmed these observations. One-way repeated measures ANOVAs showed a highly significant Time effect in both the control and treated groups (*p* < 0.001 and *p* < 0.01, respectively). Post hoc Holm-Bonferroni-corrected t-tests comparing each time point to baseline showed significantly reduced performance at days 14 and 21 in the control group (*p* = 0.045 and 0.027, respectively), but no time point reached statistical significance in the TRAM34 group. The two-way rm-ANOVA comparing the two groups showed a highly significant between-group interaction (*p* = 0.004), reflecting the initially lower mSLT performance of the TRAM34-treated animals, subsequently outperforming the control animals from day 7 onward, with similar final performance. On post hoc Holm-Bonferroni-corrected t-tests, there was no significant between-group difference at baseline, and differences did not reach statistical significance for any specific post-MCAo time point.

The mSLT data are shown in Figure 2. The within-group repeated-measures ANOVAs revealed a significant main effect of Time in both groups, documenting that MCAo impacted sensorimotor performance in both the control and the treated group (*p* < 0.001 and < 0.01, F = 5.45 and 4.65, respectively). Thus, although at baseline both groups spent almost the entire observation period attending to the stimulus, after MCAo the mSLT performance initially declined in both groups, and then recovered until the end of the experiment.

The between-group repeated-measures ANOVA revealed a significant Group × Time interaction (*p* = 0.004), indicating a different time-course of mSLT performance in the two groups. As shown in Figure 1, although the mSLT values of the TRAM-34 group were initially lower than the control values, the values then crossed and the TRAM-34 group outperformed the control group from day 7 onward, i.e., recovered faster, with similar final performance. 

### 3.2. PET Results

**Figure 3:**
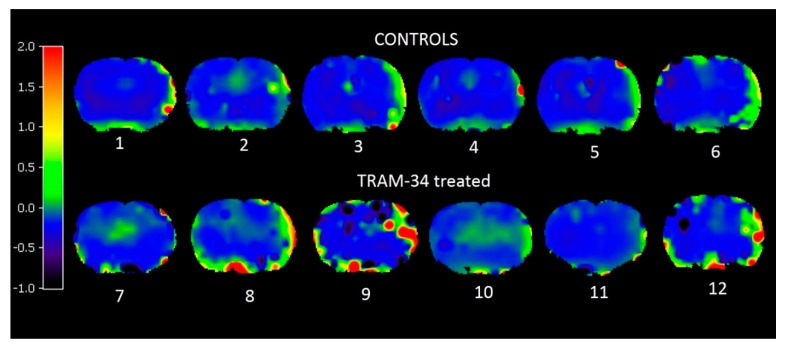
Illustrative [^11^C]-PK11195 coronal sections (one par rat, *n* = 6 rats per group). The PET images are displayed according to a pseudo-colour scale shown on the left-hand side, which ranges linearly from -1.0 to + 2.0 absolute BP_ND_ (see Methods for details, and in radiological display, i.e., the left hemispohgere is shown on the left side of the figure.). Areas with increased BP_ND_ (shown as green, yellow or red spots) affect at least part of the left cortical MCA territory in both the control and the TRAM-34 treated rats, representing abnormal microglial activation. The ‘hot’ areas at the bottom part of the images represent high uptake in extra-cerebral structures located at the base of the skull. Hot spots located inside the ventricles are also present in a few rats; they represent high [^11^C]-PK11195 binding by choroid plexus.

MRI lesions were identified in no subject from either group. Figure 3 shows representative coronal BP_ND_ parametric maps for the 12 rats of this study, showing areas of increased PK11195 binding in the affected hemisphere in nearly all rats of both groups on day 14.

Table 1 shows the findings from the within-subject statistical analyses. Significant increases in PK11195 BP_ND_ were present in the affected as compared to the unaffected hemisphere in 5/6 rats of each group. There was no significant difference in (affected–unaffected) hemisphere weighted means between the control and TRAM-34 groups (Table 2).

### 3.3. Immunofluorescence Results

**Figure 4:**
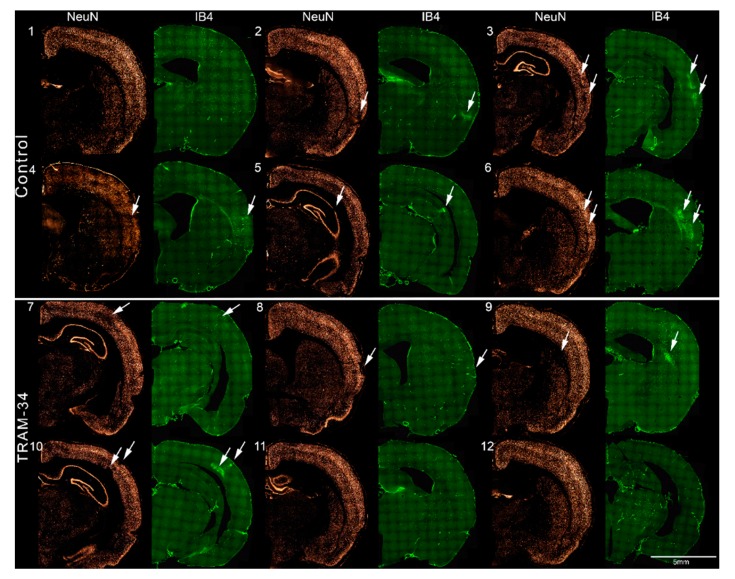
NeuN and IB4 immunofluorescence (IF) coronal sections from each rat (*N* = 6 per group) obtained at day 28 post MCA occlusion, illustrating the presence of patchy selective neuronal loss and microglial activation (NeuN and IB4, respectively) affecting the left MCA territory (arrows). Control rats and TRAM-34-treated rats are shown the top and bottom halves of the figure. See Methods for technical details on how these images were created. They illustrate (i) the excellent topographical congruence between areas with loss of NeuN and increased IB4 staining in most—though not all—cases; and (ii) the smaller extent of NeuN lesions in the TRAM-34-treated group. Please note that in some subjects, the changes in IB4 do not clearly appear in these low-magnification images, but were present on magnification (not shown). Please note that in rats 5 and 9, SNL and MA affected the dorsal hippocampus and dorsal striatum, respectively, which are part of the MCA territory in some individuals [21,23,33].

**Figure 5:**
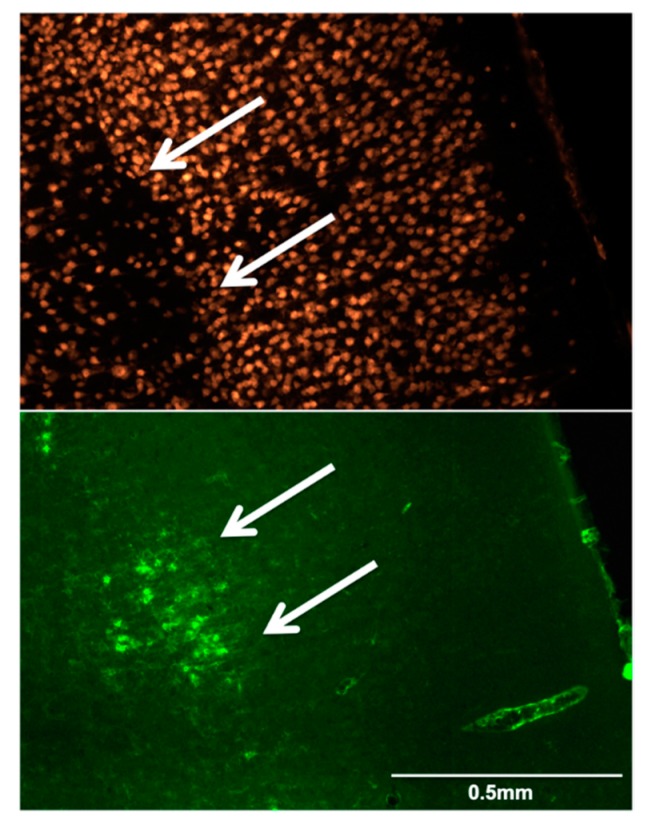
Representative high-power NeuN (top) and IB4 (bottom) immunofluorescence appearances of selective neuronal loss and microglial activation (arrows) from rat 3 (control group). Note in the IB4 image the typical appearance of activated microglia, namely ovoid or circular particles with only occasional slight ramifications.

Following sacrifice on day 28, two independent assessors, who were blinded to the animals’ group and stroke side, assessed randomly presented brain sections and determined volumes of SNL and MA based on NeuN and IB4 staining. Visual assessment of the stained sections revealed no areas of infarction in any rat, but the presence of topographically largely congruent patches of NeuN staining loss and high IB4 staining, of variable severity but exclusively within the affected-side MCA territory, which were present in 5/6 control animals and 4/6 TRAM34-treated subjects. Across rats and sections, the degree of SNL was milder in the TRAM-34-treated group. These findings are illustrated for one representative section in each rat in Figure 4. Figure 5 illustrates typical high-power appearances of selective neuronal loss and microglial activation as labelled with NeuN and IB4, respectively.

The results of the within-subject t-tests assessing the affected vs. unaffected hemisphere changes in NeuN and IB4 cell count in both groups of rats are shown in Table 1. In the controls, 3/6 and 2/6 rats had individually significant NeuN reductions and IB4 increases, respectively, with trends (*p* < 0.10) in the same direction in 1 and 2 rats, respectively. In the TRAM34-treated group, the corresponding figures were 2/6 and 5/6, with a trend for NeuN in a further rat.

There was a significant between-group difference in (affected–unaffected) hemisphere weighted-means for NeuN cell count (−2.32 ± 1.4% and 1.68 ± 2.36%, respectively; *p* = 0.009, Mann-Whitney), illustrating improved neuronal survival in TRAM-34-treated subjects. The corresponding value for IB4 was smaller in the TRAM-34 than in the control group, but the difference was not statistically significant. The data are presented in Table 2. 

## 4. Discussion

Here we tested the feasibility of a protocol designed to assess the effects of a pharmacological agent on post-MCAo selective neuronal loss (SNL), microglial activation (MA) and sensorimotor deficit (SMD) in rodents. Important features of this protocol are (i) all of the variables are measured in the same subjects; (ii) MA is assessed at two time points in the same animal, by means of in vivo PET at day 14 and post-mortem IF at day 28, respectively; and (iii) both in vivo- and post mortem-determined MA, as well as SNL, were measured in the same ROIs, using a fixed cytoarchitectonic template covering the whole MCA territory. Additional positive features of this protocol include the within-subject assessment of the effects of MCAo across the set of ROIs (see Table 1), and the enhanced power afforded by the longitudinal design as compared to studying different sets of subjects. 

Previous experimental stroke studies have used single or multiple PET scanning sessions using ligands mapping MA followed by post-mortem assessment to evaluate the effects of a pharmacological agent [52,53,54,55,56]. However, none has implemented quantitation of both in vivo and post mortem MA and SNL in the same ROIs covering the affected hemisphere in a systematic fashion, together with the longitudinal assessment of sensorimotor performance.

As a proof-of-principle study, this protocol was applied here to test the hypothesis that MA contributes to the severity of post-MCAo SNL—viz, the neurotoxic hypothesis of early MA (see Introduction)—and in turn to the degree of post-stroke SMD. The KCa3.1 blocker TRAM-34 was administered 12 h post stroke onset, a clinically meaningful scenario, and drug administration continued for 4 weeks. We found that this agent significantly curbed SNL and hastened sensorimotor recovery. These findings are consistent with a previous study using the selective COX2 inhibitor celecoxib [57], with the difference that these authors assessed SNL in cortical areas surrounding established infarcts following considerably longer—namely, 1 h—proximal MCAo. At variance with this latter study, however, the beneficial effects of TRAM-34 on SNL and SMD took place with no, or only a weak, trend of reduction of MA as assessed with in vivo [^11^C]-PK11195 PET at 14 days and IB4 IF at 28 days, respectively. TRAM-34 is a brain-penetrant small molecule that targets the calcium-activated potassium channel KCa3.1 [24,29]. TRAM-34 has been widely used as a pharmacological tool to explore the physiological and pathophysiological role of its target channel in the immune system [58]. Of relevance for this study, KCa3.1 inhibition with TRAM-34 has been shown to inhibit IL-1β, TNF-α and NO production by cultured microglia [27]. In rodent MCAo, pharmacological inhibition of KCa3.1 with TRAM-34 or genetic knockout reduces microglia activation and inflammatory cytokine production in the brain [28,29]. In Wistar rats subjected to 90 min of MCAo followed by 7 days of reperfusion twice daily, TRAM-34 treatment at 10 and 40 mg/kg started 12 h after reperfusion reduced infarct area and MA as determined by CD68 staining by ~50% while increasing neuronal survival and significantly improving neurological deficit score starting 4 days after the insult [29]. In C57BL/6J mice both genetic deletion of KCa3.1 and treatment with TRAM-34 reduced NeuN-negative infarct area and MA activation determined by Iba1 staining intensity by ~50%, and improved neurological deficit following 60 min of MCAo with 8 days of reperfusion [28]. Confirming TRAM-34’s selectivity for KCa3.1, treatment of KCa3.1^−/−^ mice following MCAo did not result in any further improvement in neurological deficit or reduction in infarction [28].

The lack of a significant TRAM-34 effect on MA in this study is somewhat unexpected, as the previous experimental stroke studies using TRAM-34 described above have reported an inhibition of MA [28,29]. However, in the current study, MA was assessed at 2 relatively late time points, so it cannot be ruled out that MA was effectively reduced at an earlier time point, at which microglia are mainly of the neurotoxic, as opposed to pro-repair, type (see Introduction). We chose 14 days for the PET study as this is a time point at which MA is around its peak in rodents after MCAo, and is therefore easily quantifiable [55,56,59,60,61,62,63]. Regarding the post-mortem assessment, although day 28 is on the downhill slope for MA [55,56,59,60,61,62,63], it is considered optimal for the assessment of SNL [19,20]. In the previous studies with TRAM-34 [28,29], MA was assessed earlier (at 7 days), under more severe conditions (60- and 90-min, instead of 15-min, MCAo), and using different activation markers (Iba1 and CD68 instead of IB4 and TSPO here). It is, therefore, conceivable that we here partly “missed” the effect of KCa3.1 blockade, or, alternatively, that IB4 expression is not reduced by KCa3.1 inhibition. 

This study suggests that blocking KCa3.1, and thus presumably inhibiting early MA, may at least in part prevent SNL in the salvaged penumbra. SNL has been directly documented using NeuN immunohistochemistry after stroke in rodents, and indirectly in man using in vivo neuronal markers (see Introduction). So far, three main mechanisms for SNL following brief MCA occlusion have been considered. First, SNL may represent delayed cell death triggered during the ischemic episode. In favour of this mechanism stand the significant correlation between severity of SNL and degree of intra-occlusion hypoperfusion, both in rats [21] and humans [12], and the fact that neuronal damage was observed in rodents as early as 24 h post-stroke [22]. The second potential mechanism for SNL is scattered neuronal death nearby occluded capillaries despite overall effective reperfusion, i.e., local ‘no-reflow’ due to pericyte contraction [64]. Thus far, no definite data in support of this eventuality is available. According to the third mechanism, which was tested in the present study, SNL may result at least in part from the well-established neurotoxic effects of activated microglia. In support of this mechanism stand the apparent concomitant progression of striatal SNL and MA observed over 3–4 weeks following brief proximal MCAo in rodents [22,65,66] (see Baron et al. [7] for review), and the striking topographic congruence between patches of SNL and MA [9,19,20,21,22] which, however, may merely reflect the initial triggering of MA by injured neurons. Regarding timing, it appears that MA in fact covers two overlapping phases, an initial one, where activated microglia are mainly neurotoxic, and a subsequent one starting at around day 7 in rodents, where microglia shift to mainly pro-repair effects [17]. Please note that in the present study, TRAM-34 was administered from 12 h after stroke onset, i.e., within the initial, primarily neurotoxic phase, and continued out to day 28. 

This study has limitations. First, the co-registration between the coronal sections from Paxinos’ stereotaxic atlas and the MRI (and then onto PET) was carried out manually, using anatomical landmarks. This methodological point has been extensively discussed in our earlier articles [19,21,23], and was chosen because no validated automatic method to co-register histopathological sections to in vivo imaging is available yet, given the major differences in slice thickness and spatial resolution. Although not optimal, we estimate the errors to be minimal given the very prominent anatomical landmarks present in the coronal sections of the Paxinos Atlas, and readily visible on the MR coronal cuts. Nevertheless, in the future, an automated and accurate method would potentially reduce such errors. In contrast, matching the histopathological coronal sections to the corresponding sections of Paxinos’ stereotaxic atlas is straightforward, given the prominent anatomical landmarks such as the hippocampus. Second, we stained histopathological sections using IB4, a reliable and widely used marker for activated microglia [47,48,49]. However, IB4 also binds to endothelial and perivascular cells, which might have been erroneously identified as activated microglial cells during the automatic assessment, despite using stringent microglial-specific morphological selection criteria, including particle size and circularity (see Methods). This might have contributed to the lack of significant effect of TRAM-34 on MA in the present study. Third, the small sample size used in this proof-of-concept investigation could have caused both Type I and Type II errors. Ligand PET studies are highly cumbersome and resource-consuming, and typically only small samples can be investigated using this in vivo imaging technique. Note, however, that the findings with MA, SNL and SMD, statistically significant or not, were consistently in the biologically expected direction, and concurred with the working hypothesis. The small sample size may also explain the lack of statistical significance for some individual time-points of the mSLT dataset, but the *p*-values were corrected for multiple tests using the post hoc Holmes-Bonferroni test, which is notoriously conservative. In the same vein, we did not use sham-operated groups because of the costs involved. The non-ischemic hemisphere was used instead as reference in the IF quantitative approaches because close inspection of the IF sections by two observers blinded to the side of the MCAo did not detect any MA or SNL in any rat (data not shown). Fourth, we used adult SHRs as this strain more closely mimics the typical stroke population, but different results might emerge with other rat strains or with aged rats. In addition, fifth, we used only the mSLT to assess sensorimotor outcome, and it would be of interest in future studies to use additional sensorimotor tasks, such as total locomotor activity and rotarod performance, as well as cognitive tasks [7]. We chose to use the mSLT because this test is highly sensitive to even mild ischemic damage—including isolated SNL—in the sensorimotor cortex [10,67], which sits in the centre of the cortical MCA territory targeted by our distal MCAo model [21]. 

## 5. Conclusions

Our findings demonstrate the feasibility and utility of our longitudinal protocol designed to test the effects of a pharmacological agent on SNL, MA and SMD after temporary focal cerebral ischemia in the rodent. As applied here to post-stroke neuroinflammation, the findings suggest that administration of the KCa3.1 inhibitor TRAM-34 starting 12 h after the ischemic insult and continued for 4 weeks reduces the amount of SNL and hastens recovery from sensorimotor deficit without a marked effect on MA, as assessed here using in vivo [^11^C]-PK11195 PET at day 14 and IB4 immunofluorescence at day 28, which may not tell the whole story regarding post-stroke neuroinflammation. These findings will need to be confirmed using larger samples, and expanded by administering microglia inhibitors in different time windows.

## Figures and Tables

**Table 1:** Results of within-subject t-tests testing the affected vs. unaffected hemisphere changes in [^11^C]-PK11195, NeuN and IB4. Two-tailed *p*-values are shown for each rat and each variable.

Rat #	PK11195 PET	NeuN	IB4
**Controls**			
1	<0.001	0.052	0.57
2	<0.001	<0.001	0.44
3	<0.001	0.41	0.09
4	<0.001	0.53	0.08
5	<0.001	0.006	0.007
6	0.266	0.036	0.014
**TRAM-34 treated**			
7	0.099	0.012	<0.001
8	<0.001	0.25	0.29
9	<0.001	0.88	0.031
10	<0.001	0.20	0.02
11	0.029	0.11	0.002
12	0.009	0.08	0.003

**Table 2:** (Affected–Unaffected) hemisphere weighted-mean differences for ^11^C-PK11195 BPND, NeuN cell count and IB4 cell count (see Methods). Two-tailed *p*-values for the between-group comparisons are also shown (Mann-Whitney test).

	^11^C-PK11195 PET	NeuN	IB4
**Controls** (mean ± SD)	+0.052 ± 0.015	−2.32 ± 1.4%	+2.52 ± 4.9%
**TRAM-34 treated** (mean ± SD)	+0.066 ± 0.071	+1.68 ± 2.36%	+0.08 ± 3.2%
Comparison	*p* = 1.0	*p* = 0.009	*p* = 0.337
